# The multi-kinase inhibitor TG02 induces apoptosis and blocks B-cell receptor signaling in chronic lymphocytic leukemia through dual mechanisms of action

**DOI:** 10.1038/s41408-021-00436-0

**Published:** 2021-03-13

**Authors:** Rong Chen, Jennifer Tsai, Philip A. Thompson, Yuling Chen, Ping Xiong, Chaomei Liu, Francis Burrows, Mariela Sivina, Jan A. Burger, Michael J. Keating, William G. Wierda, William Plunkett

**Affiliations:** 1 Department of Experimental Therapeutics, The University of Texas M.D. Anderson Cancer Center, Houston, TX USA; 2 Tragara Pharmaceuticals, Carlsbad, CA USA; 3 Department of Leukemia, The University of Texas M.D. Anderson Cancer Center, Houston, TX USA; 4 Present Address: Department of Emergency Medicine, Yale School of Medicine, New Haven, CT USA; 5 Present Address: Kura Oncology, Inc., San Diego, CA USA

**Keywords:** Chronic lymphocytic leukaemia, Targeted therapies, Drug development

## Abstract

The constitutive activation of B-cell receptor (BCR) signaling, together with the overexpression of the Bcl-2 family anti-apoptotic proteins, represents two hallmarks of chronic lymphocytic leukemia (CLL) that drive leukemia cell proliferation and sustain their survival. TG02 is a small molecule multi-kinase inhibitor that simultaneously targets both of these facets of CLL pathogenesis. First, its inhibition of cyclin-dependent kinase 9 blocked the activation of RNA polymerase II and transcription. This led to the depletion of Mcl-1 and rapid induction of apoptosis in the primary CLL cells. This mechanism of apoptosis was independent of CLL prognostic factors or prior treatment history, but dependent on the expression of BAX and BAK. Second, TG02, which inhibits the members of the BCR signaling pathway such as Lck and Fyn, blocked BCR-crosslinking-induced activation of NF-κB and Akt, indicating abrogation of BCR signaling. Finally, the combination of TG02 and ibrutinib demonstrated moderate synergy, suggesting a future combination of TG02 with ibrutinib, or use in patients that are refractory to the BCR antagonists. Thus, the dual inhibitory activity on both the CLL survival pathway and BCR signaling identifies TG02 as a unique compound for clinical development in CLL and possibly other B cell malignancies.

## Introduction

Chronic lymphocytic leukemia (CLL) is characterized by the gradual accumulation of functionally incompetent lymphocytes in the peripheral blood^1,2^. The constitutive activation of B-cell receptor (BCR) signaling in the lymph nodes^3^, together with the overexpression of the Bcl-2 family anti-apoptotic proteins^4^ represent two hallmarks of CLL, that drive the leukemia cell proliferation and sustain their survival.

BCR is composed of membrane immunoglobulin (Ig) and associated Igα/Igβ heterodimers (CD79a/CD79b). Upon antigen binding in the lymphoid tissue, the receptors on the CLL surface aggregate, leading to phosphorylation of immunoreceptor tyrosine-based activation motifs (ITAMs) in the cytoplasmic tails of Igα/Igβ by the Src family kinases such as Lyn and Fyn. This activity recruits and activates spleen tyrosine kinase (SYK), Bruton’s tyrosine kinase (BTK), and phosphatidylinositol-3-kinase delta (PI3K delta), thus initiating multiple signaling pathways that eventually lead to CLL proliferation and prolonged survival.

Unlike CLL cells in the lymphoid tissue that are constantly renewing, the majority of the CLL cells in the peripheral blood are quiescent^5^. Defects in apoptosis sustain them from spontaneous and drug-induced apoptosis^4^. The CLL cells are “primed” to death due to genomic stress or deranged signals, but apoptosis is placed on hold by the overexpressed anti-apoptotic proteins of the Bcl-2 family^6,7^. This imbalance of apoptosis control provides an effective target for CLL therapies, manifested by the clinical success of the Bcl-2 antagonist venetoclax^8^. Mcl-1 also plays a prominent role in maintaining CLL survival. Direct inhibition of Mcl-1^9^ or specific knocking down Mcl-1 by siRNA^10^ induced apoptosis in CLL cells. We and others showed that the CDK9 inhibitors roscovitine^11^, flavopiridol^12,13^, SNS-032^14,15^, dinaciclib^16^ as well as the new agents such as voruciclib^17,18^, A-1592668^19^ and AZD4573^20^, blocked the phosphorylation of RNA pol II which inhibited transcription^21,22^. This action reduced Mcl-1 and induced apoptosis in CLL cells. The selectivity of this strategy is attributed to the rapid turn-over rate of Mcl-1^23^, mediated by multiple adenylate-uridylate-rich elements in the 3′-UTR of the transcript, as well as the two PEST sequences in the Mcl-1 protein that signal for its rapid degradation^24^. Thus, upon a transient inhibition of transcription, both Mcl-1 mRNA and protein are preferentially depleted. The critical dependence of CLL cells on Mcl-1 for survival provides a biological context for these compounds to induce apoptosis selectively in the CLL cells and spare the normal cells^6,25,26^.

Here we studied the mechanisms of action of TG02 (zotiraciclib)^27,28^, an orally available, potent inhibitor of CDK1, 2, 5, and 9 (IC_50_ of 3 to 9 nM)^29^. In primary AML cells, inhibition of CDK9 reduced RNA pol II phosphorylation, which blocked transcription and led to the depletion of Mcl-1 and XIAP and the subsequent induction of apoptosis^30^. Depletion of Mcl-1 in myeloma cells was also correlated with TG02-induced cell death^31^. These preclinical studies provided rationale for phase 1 trials of TG02 as a single agent in patients with acute leukemia and multiple myeloma (NCT01204164). TG02 was granted orphan drug designation by both the US FDA and EMA for the treatment of gliomas. It is currently being actively investigated in high-grade gliomas (NCT02942264, NCT03224104), in which CDK9 inhibition results in depletion of the key oncogenic protein MYC^32^.

The Src family members Lck and Fyn are targets of TG02 with IC_50_ values of 11 nM and 15 nM, respectively^29^. Both Lck and Fyn were reported to play important roles in the proximal steps of B cells activation. They may act among the kinases that phosphorylate the ITAM and initiate the cascade of BCR pathways.

Thus, because of the unique inhibition profile, we hypothesized that TG02 may target both of the pathogenesis aspects of CLL: by blocking Lck and Fyn, TG02 may act as an antagonist to BCR signaling and abrogate the proliferation and surviving signal in the lymphoid tissue microenvironment; by inhibiting CDK9-mediated transcription, TG02 would reduce Mcl-1 and induce apoptosis.

## Materials/subjects and methods

### Patients and cells

Samples from 84 CLL patients and 3 healthy donors were used in this study. Twelve participated in the ibrutinib arm of phase II randomized study of ibrutinib versus ibrutinib plus rituximab in patients with relapsed CLL (NCT02007044). One sample progressed on ibrutinib therapy after initial response. Median age was 65 (range, 43–84) with 54 male and 30 female patients. Their median white blood cell count was 64,600/μl (range, 6200 to 649,800/μl). The median lymphocyte percentage was 91% (range, 50–99%). Detailed patient characteristics are summarized in Supplementary Table 1. Approval was obtained from the University of Texas M. D. Anderson Cancer Center Institutional Review Board for this investigation, and all patients and donors agreed to participate and provided informed consent for use of their cells for in vitro studies. Peripheral blood samples were collected in heparin vacutainer tubes and the mononuclear cells were isolated by Ficoll density-gradient centrifugation. The isolated cells were cultured at 1 × 10^7^ cells/ml in RPMI 1640 medium containing 10% of autologous patient plasma or fetal bovine serum (FBS). We found the use of autologous patient plasma is beneficial in mimicking the in vivo conditions and enhancing CLL cell survival. This culture condition is especially relevant in preclinical studies as drugs bind differently to human or bovine proteins^13^.

The wild-type and BAX/BAK double knockout mouse embryonic fibroblast (MEF) cells were provided by Dr. John Reed^33^. They were cultured in DMEM media with high glucose, 2 mM l-glutamine, and 10% FBS. The depletion of BAX and BAK protein was confirmed by immunoblotting. The human mesenchymal cell line StromaNKtert was provided by Dr. Jan Burger from our institution and was originally purchased from the Riken Cell Bank (Japan). They were maintained in RPMI media plus 10% FBS. They were authenticated by STR DNA fingerprinting at the Characterized Cell Line Core at MDACC using the AmpFlSTR Identifiler kit (Applied Biosystems). For co-culture experiments, StromaNKtert cells were seeded the day before the addition of the CLL cells. The ratio of CLL: StromaNKtert was 100:1. All cells were free of mycoplasma, as certified by Characterized Cell Line Core Facility at MDACC using the MycoAlert kit from Lonza (Switzerland).

### Materials

TG02 was provided by Tragara Pharmaceuticals Inc. (Carlsbad, CA). SNS-032 was provided by Sunesis Pharmaceuticals Inc. (South San Francisco, CA). JAK2 inhibitor TG101348 was provided by Dr. Brian Dymock from SBIO (Singapore). Sunitinib and AC220 were purchased from Selleck Chemicals (Houston, TX). Lck inhibitor (Lcki) was purchased from EMD Millipore Bioscience (Billerica, MA). [^3^H]Uridine (50 Ci/mmol) was purchased from Moravek Biochemical Inc. (Brea, CA). Annexin V-FITC was purchased from BD Biosciences (Franklin Lakes, NJ). Propidium Iodide (PI) solution (1 mg/ml) was purchased from Sigma Aldrich Inc. (St. Louis, MO). DiOC6(3) was from Life Technology (Grand Island, NY). Goat F(AB’)2 fragment to human IgM was purchased from MP Biomedicals (Solon, OH) and was prepared as 3.5 mg/ml in H_2_O.

### Quantitation of mitochondrial membrane potential loss and cell death by flow cytometry

Change in mitochondrial membrane potential and cell death were measured using the fluorescent cation DiOC6(3), annexin V and PI staining as described in detail previously^34^. To access the viability of normal lymphocytes from healthy donors after incubation with TG02, the samples were triple stained with annexin V-FITC, mouse anti-human CD3-APC, and mouse anti-human CD19-RPE (Life Technology). The apoptotic cells (annexin V positive) were measured in subpopulations stained positive for CD3 (T cells) or CD19 (B cells).

### Plasma protein binding analysis

Human plasma protein binding of TG02 and SNS-032 was analyzed using the equilibrium dialysis method in our institutional Pharmaceutical Science Facility.

### NF-κB activity assay

The TransAM^®^ NF-κB p65 Chemi kit (Active Motif, Carlsbad, CA) was used to detect NF-κB activity. TG02 or Lcki were added 1 h prior to anti-IgM, and the CLL cells were collected 2 h later. Two microgram nuclear extracts were used for p65 activation analysis by ELISA per kit instruction. Nuclear extract from TPA and calcium ionophore activated Jurkat cells were used as positive control. The wild-type and mutated p65 consensus binding oligonucleotides were used to monitor the specificity of the analysis. The chemiluminescence was read with a Synergy2 SL Luminescence Microplate Reader (BioTek, Winooski, VT).

### Evaluation of combination effect

The combination analyses of TG02 with venetoclax or ibrutinib were performed by measuring cell death after 24 h or 48 h incubation with each drug or in combination. The combination was carried out at fixed ratio of concentrations based on the IC_50_ values of each individual compound (for venetoclax combination) or a ratio of TG02: ibrutinib at 1:5. The combination index (CI) was analyzed using the median-effect method^35^ using the CalcuSyn software (Biosoft, Cambridge, United Kingdom).

### Statistical analysis

Statistical analysis was carried out using the GraphPad Prism software (GraphPad Software, Inc., San Diego, CA). A *p* value less than 0.05 was considered to be statistically significant.

## Results

### Inhibition of CDK9 was the major contributor to the toxicity of TG02 in the CLL cells

**Fig. 1:**
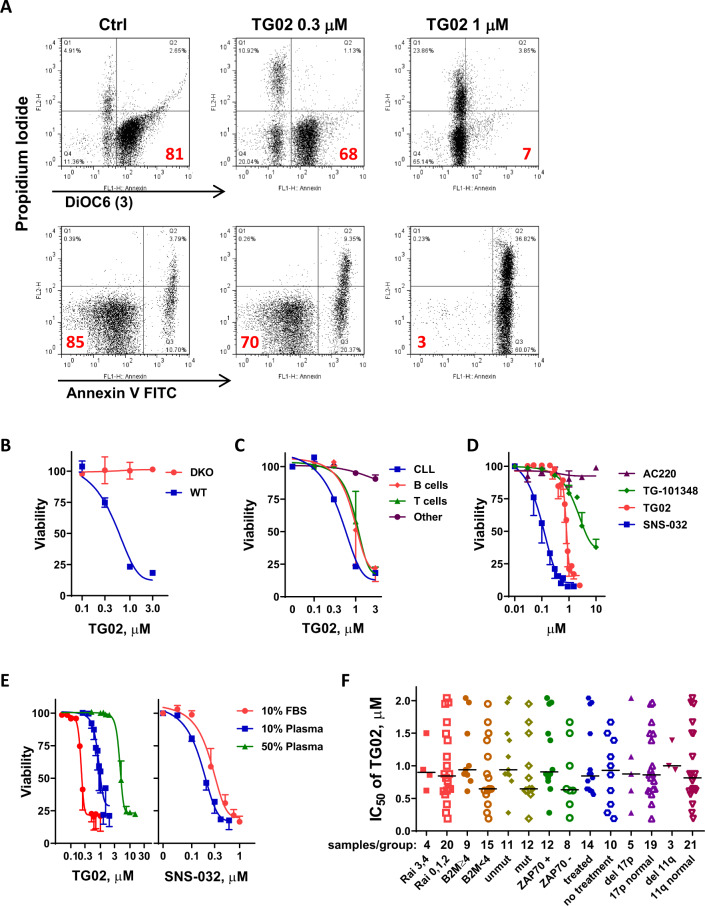
TG02-induced apoptosis in the primary CLL cells. **A** TG02-induced loss of mitochondrial membrane potential and apoptosis in the CLL cells. A representative flow image is shown. Top panel: Loss of mitochondrial membrane potential measured by DiOC6(3) and PI double staining, numbers in the lower right quadrant indicate percentage of cells that have intact mitochondrial membrane; Bottom Panel: Analysis of apoptosis by annexin V-FITC/PI double staining. The percentages of live cells (Annexin-/PI-) are shown in the lower-left quadrant. **B** TG02-induced cell death was dependent on BAX/BAK expression. The cytotoxicity of TG02 at 24 h was compared between wild-type MEF cells (◾) and cells with BAX and BAK double knockout (●). Cell death was measured by Annexin V/PI staining followed by flow cytometry and normalized to DMSO-treated controls. Data represent the mean ± SD of measurements performed in triplicates. **C** Comparison of TG02 toxicity for CLL cells relative to normal B and T cells from healthy donors. Cell death (mean ± SEM) was compared after 24 h incubation with TG02 in CLL cells (*n* = 3,◾) and normal B cells (♦), T cells (▴) cells and other cells (neither B nor T, ●) from healthy donors (*n* = 3). **D** Dose-dependent induction of cell death was compared among SNS-032 (◾, *n* = 6), TG02 (●, *n* = 10), TG-101348 (♦, *n* = 7) and AC220 (▴, *n* = 3) after 24 h incubation. Data represent the mean ± SEM. **E**. Plasma proteins reduced the potency of TG02. Left: the dose responses to TG02 at 24 h were compared in CLL cells incubated in RPMI with 10% FBS (●), 10% human plasma (◾) or 50% human plasma (▴). As a control, the dose responses to SNS-032 were compared in 10% FBS (●) or 10% human plasma (◾) (right). Data represent the mean viability ± SEM of 3 samples. **F**. Toxicity of TG02 is independent of CLL prognostic factors. IC_50_ values of TG02 after 24 h incubation were compared in 24 CLL samples with either inferior or favorable prognosis, or previous treatment history. The line in the scatter plot showed median IC_50_ values of each group. The number of samples in each group was shown below the plot. None of the comparisons was significant according to Mann–Whitney test (*p* values greater than 0.05).

We first assessed the toxicity of TG02 to the primary CLL cells. TG02 reduced the binding of lipophilic cationic dye DiOC6(3), indicating loss of mitochondrial membrane potential (Fig. 1A, top panel). This was associated with annexin V positivity (Fig. 1A, bottom panel). Compared to a dose-dependent induction of apoptosis in wild-type MEF cells, up to 3 μM TG02 did not show toxicity in BAX/BAK double knockout cells (Fig. 1B), suggesting BAX/BAK are required for TG02 to induce apoptosis. This is consistent with an intrinsic pathway of cell death. TG02 is moderately selective for CLL cells (IC_50_ 0.58 μM) compared to normal B and T cells isolated from healthy donors (IC_50_ 1.11 and 1.18 μM for B and T cells respectively) (Fig. 1C). As a multi-kinase inhibitor, TG02 potently inhibits the CDKs, as well as kinases that are well known for the pathogenesis of leukemia, such as JAK2 and FLT3^29^. To dissect their contributions to the toxicity, TG02 was compared to the CDK 2, 7, 9 inhibitor SNS-032^14,15^, the FLT3 inhibitor AC220^36^, and the JAK2 inhibitor TG-101348^37^ (Fig. 1D). A dose-response comparison showed that SNS-032 is most potent in inducing CLL cell death (IC_50_; 0.12 μM), followed by TG02 (IC_50_; 0.87 μM). AC220 at concentrations as great as 10 μM did not kill the CLL cells. TG-101348 is a weak inducer of apoptosis (IC_50_; 4.95 μM), albeit its potent inhibition against JAK2 (IC_50_, 3 nM)^37^ than TG02 (IC_50_, 19 nM for JAK2), suggesting neither FLT3 nor JAK2 contribute substantially to CLL survival. Rather, like SNS-032, inhibition of CDK9 may be a primary contributor to TG02-induced apoptosis in CLL cells.

TG02 has a comparable IC_50_ against CDK9 (3 nM) compared to SNS-032 (4 nM)^38^, but was 7 times less potent at inducing apoptosis. When the IC_50_s of TG02 were measured in CLL cells incubated in RPMI media with 10% FBS (0.24 μM), 10% human plasma (1.01 μM), or 50% human plasma (4.94 μM), we found that human plasma greatly reduced the potency of TG02 (Fig. 1E, left). This was consistent with >99% human plasma protein binding of this compound, as measured by equilibrium dialysis. In contrast, SNS-032 has greater potency when tested in 10% human plasma (0.12 μM) than in 10% FBS (0.31 μM) (Fig. 1E, right), reflecting its moderate binding (76%) to human plasma. Thus, the substantially greater plasma protein binding by TG02 may explain the discrepancy between their activities in CLL in experiments employing human plasma.

### Toxicity of TG02 is not dependent on CLL prognostic factors

Cellular and molecular markers have been identified to predict CLL disease progression or response to standard therapy containing alkylating agents and purine nucleoside analogs. For example, Rai stages 3 and 4^39^, high beta-2-microglobulin^40^, loss of TP53 or ATM loci^41^, absence of somatic IGHV gene mutation^42^, or high expression of ZAP70^43^ are predictive for aggressive disease or refractoriness to therapy. We compared IC_50_ of TG02 in 24 patient samples with either favorable or poor prognostic characteristics, or with and without prior treatment history. The median of the IC_50_ values is 0.87 μM, range from 0.19 to 2.04 μM. There were no significant differences among samples in each group (Fig. 1F), indicating that TG02 induces apoptosis by a mechanism that is independent of these prognostic factors.

### TG02 reduced the phosphorylation of RNA pol II and inhibited RNA synthesis

**Fig. 2:**
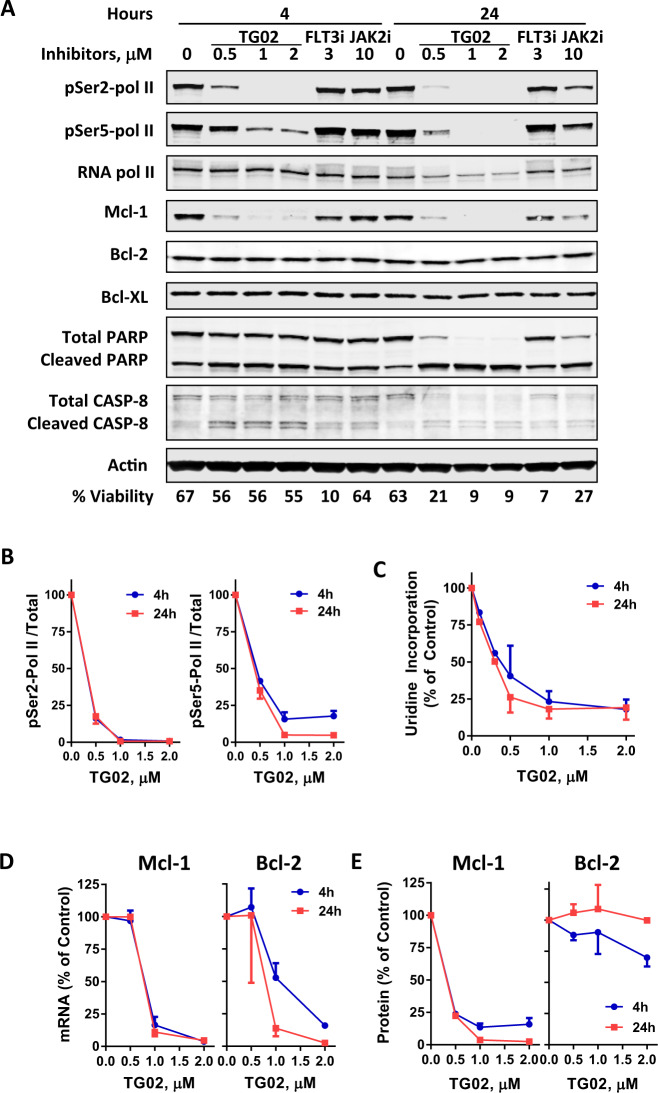
TG02 reduced the phosphorylation of RNA pol II, inhibited RNA synthesis and reduced Mcl-1 levels in CLL cells. **A** A representative immunoblot (*n* = 3) of the phosphorylation status of the Ser2 and Ser5 sites of RNA pol II CTD, Mcl-1, Bcl-2, Bcl-XL, and PARP in the CLL cells after a 4 and 24 h incubation with TG02 at 0.5, 1, and 2 μM, sunitinib (FLT3i) at 3 μM and TG-101348 (JAK2i) at 10 μM. Actin was used as a loading control. Cell viability was shown at the bottom of the blots. **B** Quantitation of the immunoblots of RNA pol II phosphorylation (Ser2, left; Ser5, right) at 4 h (●) and 24 h (◾) from 3 different CLL samples. Levels of phosphorylation were normalized to total RNA pol II and presented as percentage (mean ± SEM) of controls. **C** TG02 inhibited RNA synthesis in CLL cells. CLL cells were incubated with TG02 for 4 h (●) and 24 h (◾) and [^3^H]uridine incorporation was measured as described in “Methods”. The DPM values were normalized to time-matched controls and presented as mean ± SEM from three samples. **D** TG02 reduced the mRNAs of Mcl-1 (left) and Bcl-2 (right) in CLL cells after 4 h (●) and 24 h (◾) of incubation with TG02. **E** Quantitation of Mcl-1 and Bcl-2 proteins after 4 h (●) and 24 h (◾) of incubation with TG02 from three different CLL samples. Data were presented as percentage (mean ± SEM, *n* = 3) of controls.

Due to the quiescent nature of CLL cells in peripheral blood, we focused our studies of TG02 on inhibition of CDK9 and transcription, rather than the CDKs that regulate cell cycle progression. When CLL cells were incubated with TG02 for 4 and 24 h, there was a clear decrease in the phosphorylation of RNA pol II at the Ser2 sites of the C-terminal domain (CTD) (Fig. 2A, and quantitated in Fig. 2B), consistent with the inhibition of CDK9. To a lesser extent, the phosphorylation of Ser5 of CTD was also significantly reduced. Ser5 is the substrate of CDK7/cyclin H, which is less sensitive to TG02 than is CDK9/cyclin T (IC_50_ is 37 nM compared to 3 nM)^29^. As the consequence of RNA pol II inhibition, uridine incorporation, the measurement of RNA synthesis, was reduced dramatically in the CLL cells measured over 4 h (Fig. 2C). Neither sunitinib, a FLT3, C-kit and VEGFR inhibitor, nor TG-101348 affected RNA pol II phosphorylation (Fig. 2A).

### TG02 reduced the short-lived anti-apoptotic protein Mcl-1

The most vulnerable targets of transcription and translation inhibitors are those with short half-lives for the mRNA and protein, such as Mcl-1^21,24^. Indeed, the mRNA level of Mcl-1 was reduced by greater than 80% after a 4 h incubation with 1 μM TG02 (Fig. 2D). There was no apparent difference between 4 and 24 h. This led to rapid reduction of the protein level of Mcl-1 (Fig. 2A), quantitated in Fig. 2E. The mRNA of Bcl-2 was also reduced significantly by TG02 (Fig. 2D). However, its protein level was sustained (Fig. 2A and quantitation in 2E), consistent with its short mRNA half-life and a relatively long protein half-life^44^. There was also no apparent change in the protein level of Bcl-XL, another Bcl-2 family protein (Fig. 2A). Caspase 8 cleavage was observed within 4 h, indicating the extrinsic pathway was also activated. However, this contribution may be secondary to the intrinsic pathway as up to 3 μM TG02 did not show toxicity in BAX/BAK double knockout cells (Fig. 1B). It was reported that compounds that reduced Mcl-1 levels caused synergistic cell death when combined with the Bcl-2 antagonist venetoclax^17,19,34,45^. However, we did not see a synergistic combination effect when TG02 and venetoclax were used together (Supplementary Fig. 1). The mechanism is not clear.

### CLL sensitivity to TG02 correlated with its inhibition of CDK9 and reduction of Mcl-1

**Fig. 3:**
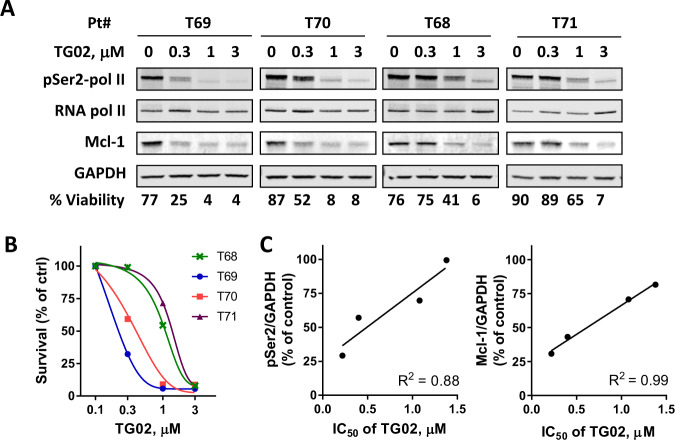
CLL sensitivity to TG02 correlated to its inhibition of CDK9 and reduction of Mcl-1. **A** Immunoblots of pSer2 and total RNA pol II, Mcl-1 after 4 h incubation with TG02 in four CLL samples that varied in their sensitivity to TG02. Cell viabilities after 24 h incubation with TG02 were shown at the bottom of the blots. **B** Dose response of the 4 CLL samples to TG02 measured at 24 h. **C** TG02 IC_50_ correlated to the inhibition of RNA pol II phosphorylation and reduction of Mcl-1. PSer2-pol II and Mcl-1 level levels (at 0.3 μM TG02) were quantitated from the immunoblots, normalized to GAPDH, calculated as percentage of controls and correlated to the IC_50_s of TG02. R squared values were generated by linear regression.

CLL cell samples varied in their sensitivity to TG02 (Fig. 1F). To investigate the mechanism of their differential susceptibility, we collected 4 samples with IC_50_s ranging from 0.22 to 1.38 μM (Fig. 3B), and compared the inhibition of RNA pol II phosphorylation at Ser2 and reduction of Mcl-1 by immunoblotting. The more responsive sample (T69) exhibited a profound reduction of both RNA pol II phosphorylation and Mcl-1 levels at 0.3 μM, compared to the less sensitive samples (T68 and T71) (Fig. 3A). High R^2^ values demonstrated linear correlations of TG02 IC_50_ to quantifications of both Ser2 phosphorylation and Mcl-1 protein level after 4 h incubation with 0.3 μM TG02 (Fig. 3C). A strong correlation was also found at 1 μM of TG02 (Supplementary Fig. 2). We previously showed that CLL cells expressing high Mcl-1 protein exhibited resistance to the translation inhibitor homoharringtonine^46^. When the expression of Mcl-1 and Bcl-2 were quantified in 14 CLL samples, we did not find a relationship between the TG02 IC_50_s to either basal Mcl-1 or Bcl-2 expression (Supplementary Fig. 3). Thus, these data suggested that CLL sensitivity to TG02 correlated to CDK9 inhibition and Mcl-1 reduction, further supporting the conclusion that inhibition of RNA pol II mediated transcription and reduction of Mcl-1 is the major mechanism of TG02-induced apoptosis in CLL cells.

### TG02 overcame stroma protection of the CLL cells

**Fig. 4:**
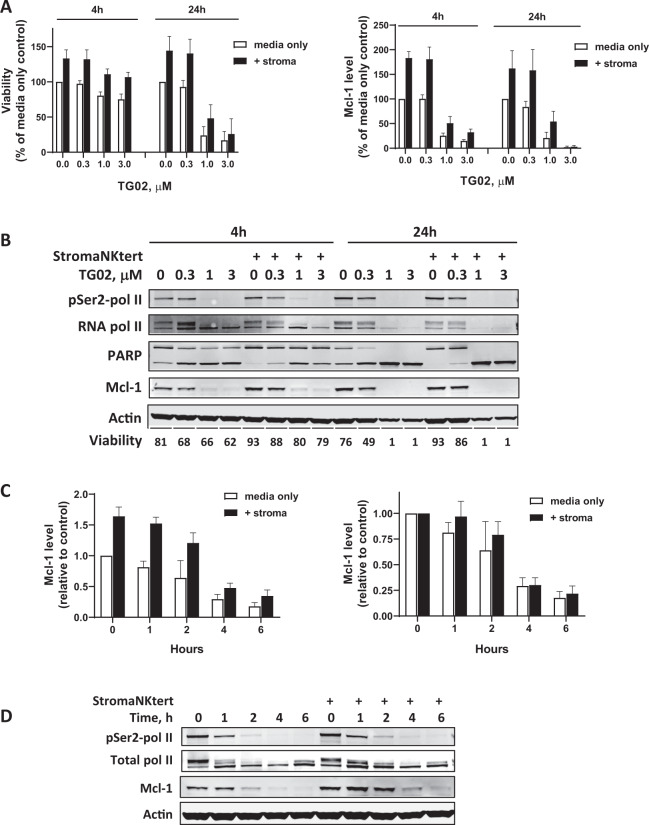
TG02 reduced Mcl-1 and induced apoptosis in the CLL cells at the presence of stroma protection. **A** TG02 overcame stroma cell protection. CLL cells were cultured in RPMI media supplemented with 10% autologous plasma, either alone (media only, white bars), or co-culture with a layer of StromaNKtert cells (+stroma, black bars) overnight. Then the cells were incubated with increasing concentrations of TG02 and viabilities were analyzed by Annexin V/PI double staining after 4 and 24 h incubation (**left**), and Mcl-1 expression were quantitated by immunoblotting (**right**). Data present percentage of media only controls (mean ± SEM) of four individual CLL samples. **B** A representative immunoblot of cells incubated in conditions described in A. **C** Mcl-1 expression was induced by co-culturing with the StromaNKtert cells, which was reduced by TG02. CLL cells were cultured in RPMI media supplemented with 10% autologous plasma, either alone (media only, white bars), or co-culture with a layer of StromaNKtert cells (+stroma, black bars) overnight. Then the cells were incubated 1 μM TG02. Cell pellets were collected at 0, 1, 2, 4, and 6 h. Phosphorylation of RNA pol II and expression of Mcl-1 were analyzed by immunoblotting. Mcl-1 levels were quantitated in these samples and presented as levels relevant to media only control (**left**) and relevant to untreated controls (**right**) (mean ± SEM, *n* = 4). **D** A representative immunoblot of cells incubated in conditions described in (**C**).

To evaluate if TG02 remains active under the protective environments in vivo^47^, we cultured the CLL cells on top of a layer of the StromaNKtert cells, which were used as a supportive feeder to mimic the bone marrow condition. There was an average of 33% increase in cell viability after a 24 h incubation with the StromaNKtert cells, compared to CLL cells alone (Fig. 4A, left). TG02 induced a dose-dependent cell death in the CLL cells after both 4 and 24 h, regardless of the protection factors. These studies were carried out in media supplemented with 10% autologous plasma. Similar results were observed when we used FBS to supplement the media, except that TG02 appeared more potent (Supplementary Fig. 4). Incubating with the StromaNKtert cells activated transcription, demonstrated by the increased phosphorylation of RNA pol II at Ser2 sites, that was associated with increased Mcl-1 expression (Fig. 4A, right and Fig. 4B). These activations were effectively blocked by TG02 in a dose-dependent manner. Although stroma induced an average of 83% increase of Mcl-1 protein compared to media only controls (Fig. 4C, D), a detailed time course showed a complete reduction of both RNA pol II phosphorylation and Mcl-1 protein within 4 h, despite a slight delay in decreasing Mcl-1 proteins at 1 and 2 h, when graphed using the untreated sample as control (Fig. 4C right).

### TG02 blocked BCR signaling in the CLL cells

**Fig. 5:**
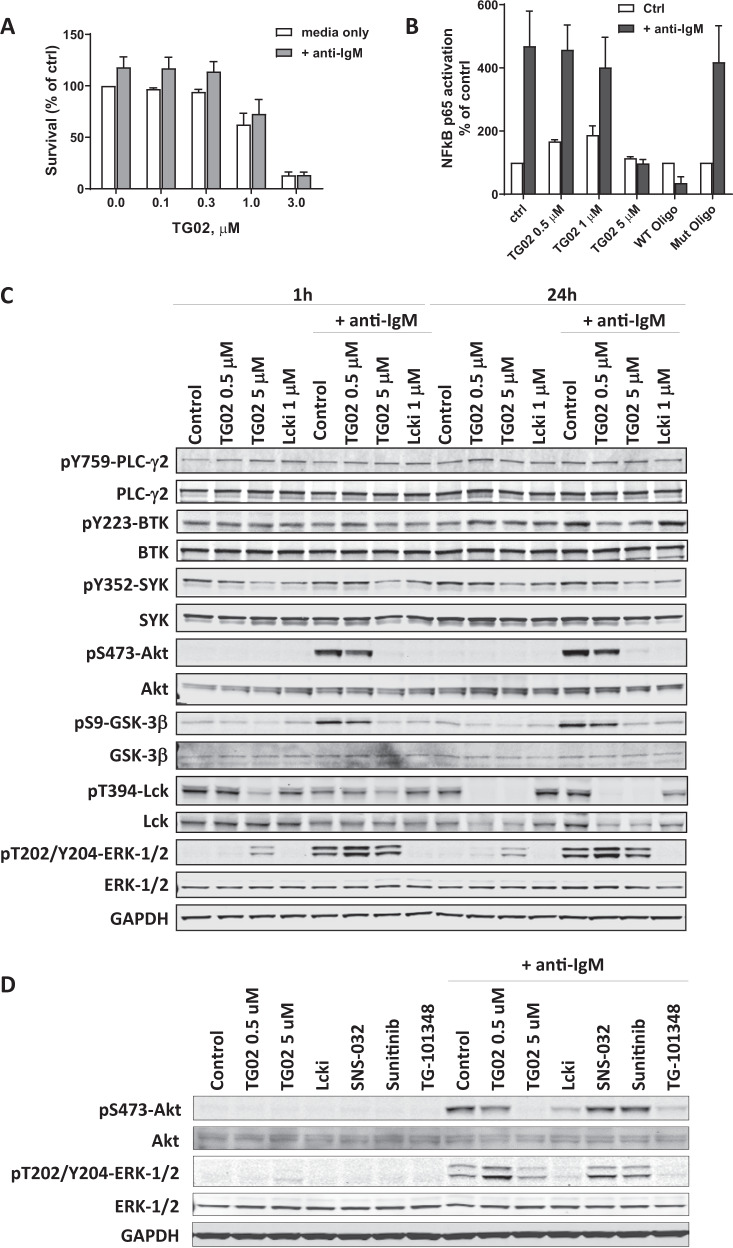
TG02 blocked BCR signaling in the CLL cells. **A** CLL cells were cultured in RPMI media supplemented with 10% autologous plasma, either alone or at the presence of anti-IgM. The cells were incubated with increasing concentrations of TG02 and viabilities were analyzed by Annexin V/PI double staining at the end of the 24 h incubation. Data represents mean ± SE of four CLL samples. **B** TG02 blocked BCR signaling-mediated activation of NF-κB. CLL cells were incubated with TG02 for 1 h before stimulating with anti-IgM for 2 h, and NF-κB p65 activation and inhibition were measured by chemiluminescence and presented as the percentage of control (mean ± SEM) of three samples. Black bars: with anti-IgM stimulation; white bars: no stimulation. WT Oligo and Mut Oligo represent the wild-type and mutated p65 consensus binding oligonucleotides that were used to confirm the specificity of the analysis. **C** The phosphorylation status of kinases in the BCR signaling pathway was analyzed by immunoblotting. GAPDH was used as loading control. CLL cells were incubated with TG02 or Lcki for 1 h before stimulating with anti-IgM for 1 h and 24 h. A representative immunoblot of three experiments is shown. **D** Inhibition of BCR signaling by TG02 was mediated by the inhibition of Lck and JAK2. CLL cells were incubated with TG02, Lcki, SNS-032, Sunitinib, or TG-101348 for 1 h before stimulating with anti-IgM for 1 h. The phosphorylation of Akt and ERK was evaluated by immunoblotting. A representative immunoblot of three experiments is shown.

To evaluate the effect of TG02 under the protective environments in lymph nodes, we cultured the CLL cells with goat F(ab’)_2_ fragment to human IgM (anti-IgM) to crosslink BCR to mimic its in vivo activation in lymphoid tissue. Incubation with anti-IgM increased the cell viability by an average of 18% after 24 h, compared to CLL cells alone (Fig. 5A), which was reduced by TG02 in a dose-dependent manner.

We further extended investigations of TG02 to BCR signaling based on its inhibitory activity against Lck and Fyn. NF-κB was activated by anti-IgM, measured by a chemiluminescence assay; this was blocked in a concentration-dependent manner by TG02 (Fig. 5B). Immunoblots demonstrated constitutive auto-phosphorylation of Lck at Thr394 and constitutive phosphorylation of SYK at Tyr352 in the CLL samples (Fig. 5C). This was reduced by TG02 in a dose-dependent manner. BCR crosslinking by anti-IgM activated Akt, represented by the phosphorylation at Ser473, as soon as 1 h after anti-IgM addition (Fig. 5C, D), and persisted for 24 h in some samples. Multiple pathways downstream of BCR signaling may lead to Akt activation. It can be activated by the PI3 kinase, or through NF-κB^48^. Akt phosphorylates and inactivates GSK-3β, thus removing the degradation signal of Mcl-1, leading to Mcl-1 stabilization^49^. Immunoblots showed that both the phosphorylation of Akt and GSK-3β were reduced by TG02 as well as by Lcki, a specific inhibitor for Lck^50^. TG-101348, but not SNS-032 nor sunitinib, also inhibited Akt phosphorylation (Fig. 5D), indicating inhibition of JAK2 by TG02 may also contributed to Akt inhibition. ERK activation was apparent after the addition of anti-IgM, and was blocked by Lcki, but interestingly not by TG02 (Fig. 5C). Rather, there was a clear induction of ERK phosphorylation by TG02. This may be associated with the inhibition of the CDKs, as induction of ERK phosphorylation was also seen with SNS-032 (Fig. 5D). Both BTK and its downstream target PLC-γ2 were constitutively activated in CLL (Fig. 5C). Neither TG02 nor Lcki affected their phosphorylation status. We did not pursue the effect of Fyn inhibition due to the lack of a specific Fyn inhibitor.

**Fig. 6:**
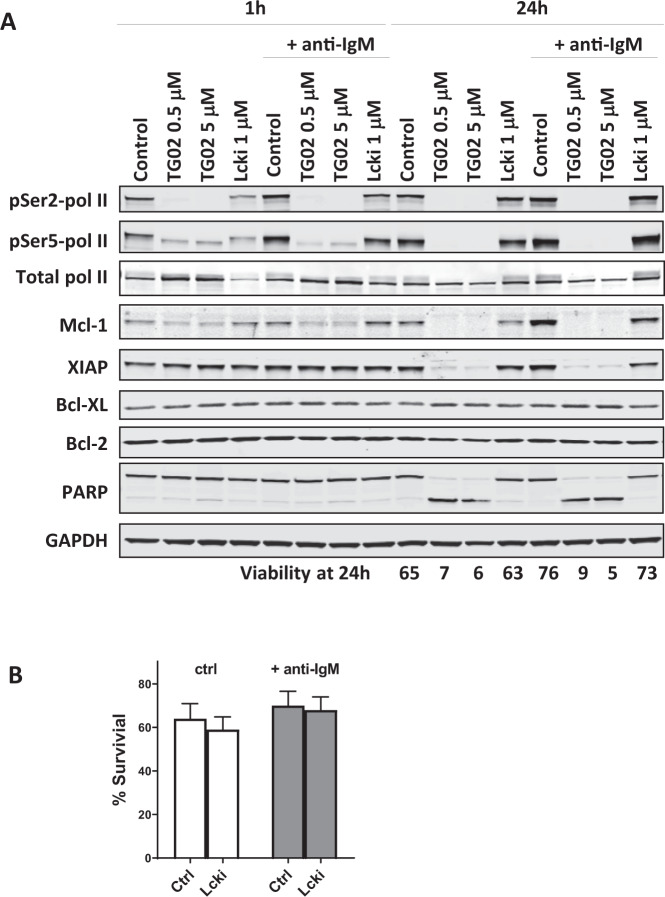
BCR signaling activated RNA pol II and increased Mcl-1 levels; this was blocked by TG02. **A** CLL cells were incubated with TG02 or Lcki for 1 h before stimulating with anti-IgM for 1 h and 24 h. The phosphorylation RNA pol II and the levels of anti-apoptotic proteins were evaluated by immunoblotting. The mean CLL cell viability of three individual experiments assessed at 24 h is shown below the image. **B** Inhibiting Lck had minimal toxicity on the CLL cells. CLL cells were incubated with Lcki for 1 h before stimulating with anti-IgM for 24 h. The percentage of surviving cells with or without Lcki was compared in groups without (white bars) and with (gray bars) anti-IgM stimulation (mean ± SEM, *n* = 8).

Immunoblots showed that anti-IgM activated transcription, shown by the enhanced phosphorylation at both the Ser2 and Ser5 sites of the CTD (Fig. 6A), together with the induction of Mcl-1 and XIAP. All these were effectively reversed by TG02. Lcki also blocked BCR-induced Mcl-1 and XIAP, likely acting through the Akt/GSK-3β pathway. However, Lcki alone was not sufficient to induce substantial apoptosis (Fig. 6B), indicating that the inhibition of Lck/Akt alone may not lower Mcl-1 sufficiently to reach the threshold to induce cell death. Rather, reducing Mcl-1 through transcription inhibition is likely the major mechanism of apoptosis by TG02, and this action may be facilitated by the inhibition of BCR signaling.

### CLL cells after ibrutinib treatment remain sensitive to TG02

**Fig. 7:**
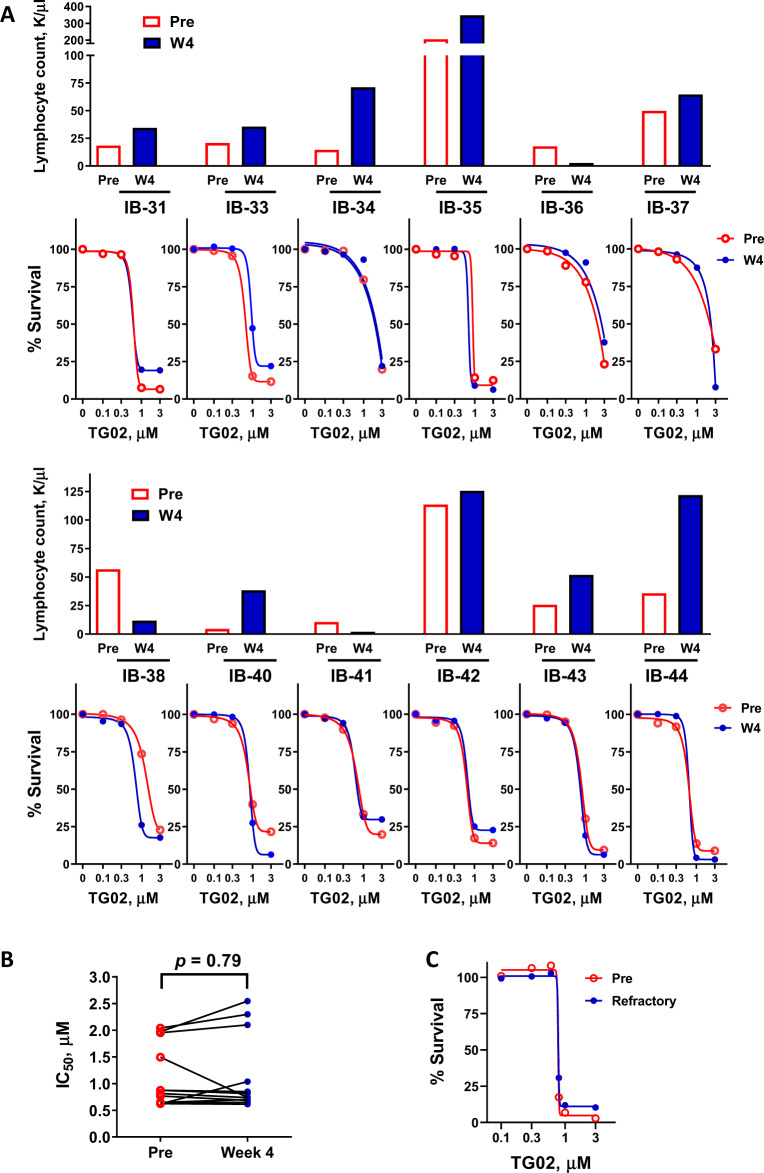
CLL cells after ibrutinib treatment remain sensitive to TG02. **A** (**top**) The lymphocyte count of the CLL patient before (Pre) and 4 weeks after (W4) ibrutinib treatment. **A** (**bottom**) Dose response to TG02 in CLL cells pre- and post- ibrutinib treatment. CLL cells isolated from CLL patients pre- (Pre, ○) and 4 weeks (W4, •) post ibrutinib were incubated for 24 h with TG02, and cell death was measured by flow cytometry. **B** The IC_50_ values of TG02 pre- (○) and 4 weeks (•) post ibrutinib were compared by Wilcoxon paired test (*n* = 12). **C** Dose response to TG02 in cells from a CLL patient collected prior to ibrutinib treatment (Pre) and at the time of refractory (Refractory).

**Fig. 8:**
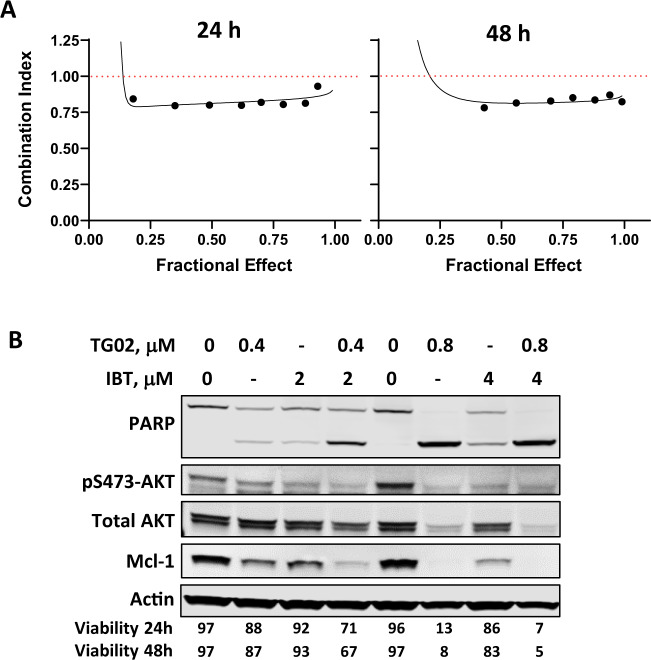
Moderate synergy in the combination of TG02 and ibrutinib. **A** Representative median-effect cures of the combination of TG02 and ibrutinib at 24 h (left) and 48 h (right). Cells were incubated with TG02 and ibrutinib at a fixed ratio of 1:5. The representative results from 4 CLL samples were shown. **B** A representative immunoblot of the combinations of TG02 and ibrutinib (IBT) at two different doses. Cell viability at 24 and 48 h were shown below the blots.

To investigate if the CLL cells are sensitive to TG02 during the lymphocytosis phase after ibrutinib treatment, a dose response to TG02 was measured in CLL cells pre- and 4 weeks post ibrutinib, when lymphocytosis was reported to be most prominent^51^. Nine of the twelve patients showed an increased lymphocyte count at the time of sampling, indicating lymphocytosis (Fig. 7A). All samples were similarly responsive to TG02 (Fig. 7B). The IC_50_ values were 1.11 ± 0.17 and 1.15 ± 0.21 μM (mean ± SEM) for the pre- and post-ibrutinib samples, respectively. Thus, the CLL cells liberated from the lymphatic tissue during lymphocytosis remain sensitive to TG02. To evaluate if TG02 is effective in CLL cells that are refractory to ibrutinib, we collected a pair of pre-treatment and ibrutinib resistant cells from the same CLL patient, whose disease progressed after three years on ibrutinib. Targeted sequencing detected C481S mutation in BTK. Both samples responded similarly to TG02 (Fig. 7C). Detailed studies confirmed that the refractory sample was less responsive to ibrutinib-induced toxicity, as well as reduction of BTK and PLCγ2 phosphorylation (Supplementary Fig. 5). Although ibrutinib alone did not lower Mcl-1 level sufficiently to induce >10% cell death, we hypothesized that it might supplement the Mcl-1 reduction by TG02 and enhance its toxicity. A median-effect analysis confirmed moderate synergy when TG02 and ibrutinib were combined at both 24 and 48 h (Fig. 8A). An immunoblot demonstrates reduction of AKT phosphorylation and Mcl-1 protein by ibrutinib, which was associated with more reduction of Mcl-1 and better than additive induction of apoptosis when combined with TG02, shown by PARP cleavage and cell viability measurement by flow cytometry (Fig. 8B).

## Discussion

This study investigated the mechanism of actions of the multi-kinase inhibitor TG02 in CLL. The results supported the hypothesis that TG02 may target both of the key elements of the pathogenesis of CLL: the deregulated BCR signaling in the lymphoid tissue and the overexpression of the anti-apoptotic proteins that sustain CLL cell viability. Among the multiple targets, inhibiting CDK9 contributed mainly to its cytotoxicity, compared to the inhibition of FLT3, JAK2, or the Src family kinases. By inhibiting CDK9, TG02 blocked RNA pol II-mediated transcription, markedly reduced the level of the short-lived anti-apoptotic protein Mcl-1, and induced robust apoptosis in the CLL cells. This mitochondrial pathway of cell death was dependent on BAX and BAK and was independent of the common prognostic factors in CLL. In addition, through the inhibition of Lck and Fyn, TG02 reversed the BCR-mediated activation of NF-κB, SYK, and Akt, an action to abrogate the protection of CLL cells by the microenvironment.

Both Fyn and Lck are heavily involved in BCR signaling. Upon antigen engagement, Fyn is one of the major Src family members to phosphorylate ITAM of the Igα/Igβ and initiate the signaling cascade^52,53^. Lck was reported to be highly expressed in CLL cells^50^. Inhibition of Lck blocked BCR crosslinking-induced phosphorylation of CD79a, as well as downstream kinases such as SYK, IKK, Akt, and ERK, suggesting that Lck also mediates a proximal signaling event^50^. We demonstrated a reduction of phosphorylation of Lck, SYK, Akt, and NF-kB by TG02 (Fig. 5), consistent with interfering at the early stages of BCR signaling, likely through the inhibition of Lck and Fyn. In addition, Fyn and Lck are well known as the initiating kinases in T-cell receptor signaling that promote T cell activation and differentiation. Thus, inhibition of Fyn and Lck by TG02 likely contributes to its toxicity toward normal T cells (Fig. 1C), which was not seen with other CDK inhibitors^14^.

Specific inhibition of Lck did not contribute perceptibly to the cytotoxic effect of TG02 (Fig. 5A). Similarly, although active in the clinic, ibrutinib^51^ and idelalisib^54^ do not induce significant apoptosis in vitro at clinically relevant concentrations, suggesting that BCR antagonists may not kill the CLL cells directly. The most remarkable clinical responses to ibrutinib and idelalisib were dramatic nodal shrinkage accompanied by temporary lymphocytosis, which peaked during the first four weeks of treatment, and gradually resolved over the next months^51^. Inhibiting BCR signaling may interrupt the homing and retention of the CLL cells within the lymph node, redistributing the B cells to the peripheral circulation. Prolonged lymphocytosis does not indicate a suboptimal response to therapy^55^, but provides an opportunity for combination with cytotoxic agents to eliminate the enriched circulating CLL population. This strategy was validated by the clinical success of combination of ibrutinib and venetoclax^56^. TG02 possesses the unique characteristics of targeting both BCR signaling as well as CLL survival. It may act as two individual drugs in combination. On one side, the inhibition of Lck and Fyn may diminish BCR activation, and thereby mobilization the CLL cells from the lymph nodes. On the other side, once the leukemia cells were deprived of the protective influence of the microenvironment, they are vulnerable to the cytotoxic effects of TG02. In addition, the observed synergy in the combination of TG02 and ibrutinib (Fig. 8) suggested that the BCR antagonist activity of TG02 may contribute to a portion of Mcl-1 reduction and augment the cytotoxic action of TG02. Further, our studies showed that TG02 killed the CLL cells efficiently in the presence of stroma protection or BCR crosslinking (Figs.4A, 5A), demonstrating that BCR activation or stroma does not provide substantial protection from apoptosis induced by Mcl-1 depletion. Thus, CLL cells in lymph nodes or bone marrow may be susceptible to TG02 as well. The observation that CLL cells sampled during the lymphocytosis phase or when progression on ibrutinib therapy remained responsive to TG02 (Fig. 7), together with the synergistic combination of TG02 and ibrutinib, suggested that TG02 could be combined with BCR antagonists in the clinic, or be used in CLL patients who are refractory to the BCR pathway-directed drugs.

Small molecules with potent inhibitory activity toward CDK9, including flavopiridol, roscovitine, SNS-032, and dinaciclib, have been evaluated in both preclinical and in clinical trials for the treatment of CLL^57^. These compounds share similar activities in CLL cells: inhibition of RNA pol II phosphorylation, reduction of Mcl-1 levels, and robust induction of apoptosis. While demonstrating features typical of other CDK9 inhibitors, disruption of BCR signaling through the inhibition of Lck and Fyn identified TG02 as a novel dual inhibitor with the potential to target CLL cells both in lymphatic tissue and in circulation. In addition, unlike the rapid plasma clearance of flavopiridol^58^, SNS-032^15^, and dinaciclib^59^, pharmacokinetic studies during the phase 1 trials showed that around 3 μM TG02 was sustained in plasma for up to 24 h^60^. This concentration is considerably greater than the IC_50_ value of TG02 determined in this study (0.87 μM). This may be related to the high plasma protein binding of TG02^61^. This could be another differentiating feature of TG02 as a sustained active plasma level for 8–12 h may be required to induce substantial apoptosis in the CLL cells^14^. Thus, our results strongly suggest that TG02, a small molecule with dual actions in both apoptosis control as well as BCR signaling, holds a promising utility in CLL therapy.

## Supplementary information

Supplemental Materials and Methods

Supplemental Table

Supplemental Figures
