# Supplementary material for: The multi-kinase inhibitor TG02 induces apoptosis and blocks B-cell receptor signaling in chronic lymphocytic leukemia through dual mechanisms of action
Source: Blood Cancer J. 2021 Mar 13;11(3):57. doi: 10.1038/s41408-021-00436-0 (PMC7956145; doi:10.1038/s41408-021-00436-0)
Supplement: Supplementary file 1 — Supplemental Materials and Methods [file 41408_2021_436_MOESM1_ESM.docx]

#### Supplemental Materials and Methods:

#### Measurement of RNA synthesis

RNA synthesis was measured by quantitating incorporation of [^3^H]uridine into the perchloric acid-insoluble materials. Briefly, after incubation with the compounds, CLL cells were labeled for 1 h with [^3^H]uridine (10 μCi/ml). They were then washed twice with 10 ml of ice-cold PBS and lysed with 0.4 N perchloric acid. Following centrifugation, the pellet was dissolved in 1 ml H_2_O with 50 µl of 0.5 N KOH overnight. The supernatant was transferred to scintillation vials to quantify radioactivity.

#### RNA isolation and Real-time quantitative PCR

Total cellular RNA was isolated from the primary CLL cells by QIAcube using the RNeasy mini kit (QIAGEN, Valencia, CA) with DNase digestion to completely remove the genomic DNA. Total RNA (20-50 ng) was used for the one-step real-time PCR reaction in the TaqMan^®^ One-Step RT-PCR Master Mix (Applied Biosystems, Foster City, CA). Each PCR reaction was carried out in a 25 µl volume on 96-well optical reaction plate for 30 min at 48°C for reverse transcription reaction, followed by 10 min at 95°C for initial denaturing, then followed by 40 cycles of 95°C for 15 sec and 60°C for 2 min in the 7900HT Sequence Detection System (Applied Biosystems, Foster City, CA). The relative gene expression was analyzed by the Comparative Ct method using 18s ribosomal RNA as endogenous control. All primers and probes and RT-PCR reaction buffers were purchased from Applied Biosystems.

#### Immunoblotting

CLL cells were lysed in RIPA buffer (EMD Millipore, Billerica, MA) with the addition of the cOmplete™ Mini Protease Inhibitor Cocktail Tablets and PhosSTOP™ inhibitor tablets for phosphatase (Sigma-Aldrich). Cell lysate proteins (20 µg) were separated by SDS-polyacrylamide gel electrophoresis and then electro-transferred to a nitrocellulose membrane (GE Osmonics Labstore, Minnetonka, MN). After 1 h blocking, the membranes were incubated with primary antibodies for 3 h, followed by incubation with secondary antibodies conjugated with fluorescent dyes for 1 h. The blots were scanned by an Odyssey Infrared Imaging system (LI-COR Biosciences, Lincoln, Nebraska) to obtain images and quantitations. The antibodies to Mcl-1 (S-19) and Bcl-2 (100), Bcl-XL, PLC-γ2 and Lck were purchased from Santa Cruz Biotechnology (Santa Cruz, CA). The antibody to PARP was from Biomol International Inc. (Plymouth Meeting, PA). XIAP, pY551 and total BTK antibodies were from BD Biosciences Pharmingen (San Diego, CA). Antibodies for total RNA pol II (8WG16), phosphorylated CTD at Ser2 (H5) or Ser5 (H14) were purchased from Covance Research Products, Inc. (Berkeley, CA). Antibodies to actin and pY394-Lck were from Sigma Aldrich (St. Louis, MO). Antibodies for GAPDH, pY352-SYK and total SYK, pT308, pS473-Akt and total Akt, pS9 and total GSK-3β, pT202/Y204 and total ERK1/2, pY759-PLC-γ2 pY1217-PLC-γ2, pY223-BTK were purchased from Cell Signaling (Danvers, MA). Alexa Fluor^®^ 680 goat anti-mouse IgG and IgM were purchased from Invitrogen (Carlsbad, California). IRDye 800CW Goat Anti-rabbit IgG was from LI-COR Biosciences.
