# Supplementary material for: The multi-kinase inhibitor TG02 induces apoptosis and blocks B-cell receptor signaling in chronic lymphocytic leukemia through dual mechanisms of action
Source: Blood Cancer J. 2021 Mar 13;11(3):57. doi: 10.1038/s41408-021-00436-0 (PMC7956145; doi:10.1038/s41408-021-00436-0)
Supplement: Supplementary file 2 — Supplemental Table [file 41408_2021_436_MOESM2_ESM.docx]

**Supplemental Table 1: Characteristics of the CLL patients.**

| Patient No. | Age | Gender | WBC count | IgV_H_ mutation* | ZAP70^†^ | B2M, mg/L | No. Prior treatment | TP53 del^‡^ | %ATM del^‡^ |
| --- | --- | --- | --- | --- | --- | --- | --- | --- | --- |
| 1 | 62 | M | 21 | mutated | - | 1.8 | 0 |  |  |
| 2 | 63 | M | 31 | unmut | - | 2.8 | 0 |  |  |
| 3 | 74 | M | 49 | mutated | - | 2.8 | 2 |  |  |
| 4 | 75 | M | 65 | NA | - | 3.8 | 0 |  |  |
| 5 | 64 | F | 95 | mutated | - | 4.4 | 0 |  |  |
| 6 | 76 | F | 89 | mutated | NA | 4.2 | 1 |  |  |
| 7 | 67 | M | 65 | mutated | - | 4.6 | 0 |  | 64 |
| 8 | 67 | F | 81 | unmut | + | 3.3 | 0 |  | 66 |
| 9 | 65 | F | 32 | unmut | NA | 2.2 | 0 | yes |  |
| 10 | 54 | M | 11 | mutated | - | 2.4 | 0 |  |  |
| 11 | 67 | M | 150 | unmut | NA | 4.3 | 0 |  |  |
| 12 | 50 | F | 25 | mutated | - | 1.7 | 0 |  |  |
| 13 | 52 | M | 114 | unmut | + | 3.1 | 0 | yes |  |
| 14 | 60 | F | 117 | mutated | - | 2.5 | 0 |  |  |
| 15 | 43 | F | 88 | mutated | NA | 3.2 | 0 |  |  |
| 16 | 51 | M | 111 | mutated | NA | 2.6 | 0 |  |  |
| 17 | 63 | F | 98 | mutated | - | 2.6 | 0 |  |  |
| 18 | 64 | F | 142 | NA | - | 7.4 | 0 |  | 24 |
| 19 | 53 | F | 22 | NA | NA | 1.8 | 0 |  |  |
| 20 | 67 | F | 72 | mutated | - | 2 | 0 |  |  |
| 21 | 58 | F | 166 | unmut | + | 2.9 | 0 |  |  |
| 22 | 46 | M | 59 | unmut | - | 3.7 | 1 |  |  |
| 23 | 58 | M | 73 | unmut | - | 2 | 0 |  |  |
| 24 | 63 | M | 36 | mutated | - | 2.1 | 0 |  |  |
| 25 | 51 | M | 286 | NA | NA | 8.2 | 0 | yes |  |
| 26 | 76 | M | 64 | NA | - | NA | 0 |  |  |
| 27 | 71 | M | 91 | unmut | - | 3.5 | 0 |  |  |
| 28 | 72 | M | 269 | unmut | + | 5.9 | 2 | NA | NA |
| 29 | 52 | M | 71 | NA | - | 2.8 | 0 |  |  |
| 30 | 54 | M | 91 | unmut | + | 2.7 | 1 |  |  |
| 31 | 52 | M | 130 | mutated | + | 5.1 | 0 |  |  |
| 32 | 65 | M | 48 | mutated | + | 4.4 | 0 |  |  |
| 33 | 69 | M | 33 | unmut | NA | 3.6 | 1 |  |  |
| 34 | 65 | M | 124 | NA | + | 3.7 | 1 |  | 87 |
| 35 | 84 | M | 37 | NA | - | NA | 1 |  |  |
| 36 | 64 | M | 22 | mutated | NA | 2 | 3 |  |  |
| 37 | 62 | M | 6 | unmut | + | 4.2 | 1 |  | 76 |
| 38 | 72 | M | 24 | mutated | - | 2.9 | 0 | yes |  |
| 39 | 78 | F | 23 | mutated | + | 3.2 | 3 |  |  |
| 40 | 63 | F | 214 | unmut | _+_ | 5.1 | 1 | yes |  |
| 41 | 61 | M | 25 | unmut | + | 4 | 1 |  |  |
| 42 | 58 | M | 57 | unmut | + | 4.9 | 1 | yes |  |
| 43 | 64 | M | 62 | unmut | + | 6.5 | 1 |  |  |
| 44 | 44 | M | 9 | unmut | + | 2.8 | 1 |  |  |
| 45 | 66 | M | 14 | unmut | + | 4.1 | 1 |  |  |
| 46 | 65 | M | 118 | mutated | - | 1.9 | 2 |  |  |
| 47 | 72 | M | 29 | mutated | + | 3.5 | 1 |  |  |
| 48 | 63 | F | 43 | mutated | + | 3.8 | 1 |  |  |
| 49 | 69 | M | 54 | NA | + | 6 | 1 |  | 30 |
| 50 | 71 | M | 265 | mutated | + | 3.9 | 0 |  | 39 |
| 51 | 53 | F | 22 | mutated | - | 2 | 0 |  |  |
| 52 | 73 | F | 85 | mutated | - | 3.6 | 0 |  |  |
| 53 | 67 | M | 69 | mutated | - | 4.8 | 0 |  |  |
| 54 | 74 | M | 12 | NA | - | 2.2 | 0 |  |  |
| 55 | 58 | M | 87 | unmut | + | 3.8 | 0 |  | 18 |
| 56 | 45 | M | 16 | mutated | NA | 1.4 | 0 |  |  |
| 57 | 73 | M | 66 | mutated | + | 4.4 | 3 |  |  |
| 58 | 68 | M | 50 | mutated | - | 4.3 | 0 |  |  |
| 59 | 69 | M | 25 | unmut | - | 2.3 | 0 |  |  |
| 60 | 84 | F | 31 | mutated | - | 3 | 0 |  |  |
| 61 | 64 | M | 32 | mutated | - | 1.7 | 0 |  |  |
| 62 | 74 | F | 70 | unmut | + | 6.8 | 0 |  |  |
| 63 | 60 | M | 148 | mutated | - | 2.8 | 0 |  |  |
| 64 | 74 | F | 72 | mutated | + | 1.8 | 0 |  |  |
| 65 | 60 | F | 260 | mutated | - | 2.4 | 0 |  |  |
| 66 | 69 | F | 64 | mutated | - | 1.8 | 1 |  |  |
| 67 | 69 | M | 47 | mutated | NA | 2.8 | 1 |  |  |
| 68 | 70 | F | 176 | mutated | - | 2.7 | 0 |  |  |
| 69 | 68 | M | 102 | unmut | + | 4.3 | 0 |  | 63 |
| 70 | 54 | M | 650 | unmut | + | 6.5 | 0 |  | 91 |
| 71 | 66 | F | 86 | unmut | + | 3.8 | 0 |  |  |
| 72 | 78 | F | 15 | NA | - | 3.4 | 2 |  |  |
| 73 | 58 | M | 30 | mutated | - | 1.8 | 0 |  |  |
| 74 | 78 | F | 95 | mutated | - | 2.4 | 0 |  |  |
| 75 | 71 | M | 39 | mutated | - | 2.3 | 0 |  |  |
| 76 | 56 | M | 73 | mutated | - | 2.1 | 0 |  |  |
| 77 | 45 | M | 48 | mutated | NA | 2.4 | 0 |  |  |
| 78 | 70 | F | 41 | mutated | + | 2.1 | 0 |  |  |
| 79 | 65 | F | 124 | unmut | + | 10.1 | 0 |  |  |
| 80 | 72 | M | 44 | mutated | - | 2.4 | 0 |  |  |
| 81 | 57 | M | 133 | mutated | - | 2.2 | 0 |  |  |
| 82 | 60 | M | 150 | mutated | NA | 3.8 | 0 |  |  |
| 83 | 74 | F | 92 | mutated | NA | 2.7 | 1 |  |  |
| 84 | 69 | M | 92 | unmut | + | 7.3 | 2 |  |  |

B2M: Beta-2-microglobulin; NA: information not available; -, negative; +, positive

*: IgHV gene with less than 98% homology with the corresponding germ-line gene was considered mutated.

†: ZAP-70 expression was detected with either fluorescence in situ hybridization or flow cytometry. Sample with greater than 20% of CLL cells expressing ZAP-70 is considered ZAP-70 positive.

‡: TP53 and ATM gene were detected using fluorescence in situ hybridization of bone marrow cells using fluorescent probes designed to detect the 17p13.1 (TP53 gene) region of chromosome 17 and the 11q22.3 (ATM gene) region of chromosome 11. A total of 200 interphases were analyzed for each probe. The normal cut off for MDACC Cytogenetics Laboratory is 4.5% for deletion of a TP53 gene and 5.1% for deletion of an ATM gene.
