# Supplementary material for: The multi-kinase inhibitor TG02 induces apoptosis and blocks B-cell receptor signaling in chronic lymphocytic leukemia through dual mechanisms of action
Source: Blood Cancer J. 2021 Mar 13;11(3):57. doi: 10.1038/s41408-021-00436-0 (PMC7956145; doi:10.1038/s41408-021-00436-0)
Supplement: Supplementary file 3 — Supplemental Figures [file 41408_2021_436_MOESM3_ESM.pdf]

Chen, R et al., Supplemental Fig. 1

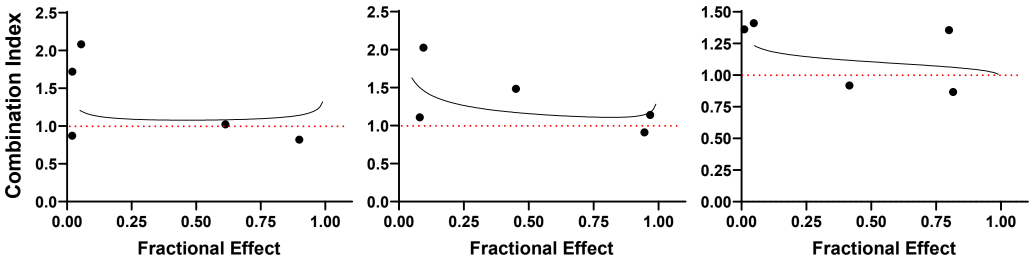

Supplemental Figure 1. Lack of synergy in the combination of TG02 and venetoclax. CLL cells were incubated with a series of concentrations of TG02 clone, venetoclax alone, or a combination of TG02 and venetoclax at a fixed ratio based on the IC_{50} values of each individual drug in three CLL samples, and cell viability at 24 h were measured by flow cytometry. The combination effects were evaluated by the median-effect analysis. The data showed the results of three individual samples.

Chen, R et al., Supplemental Fig. 2

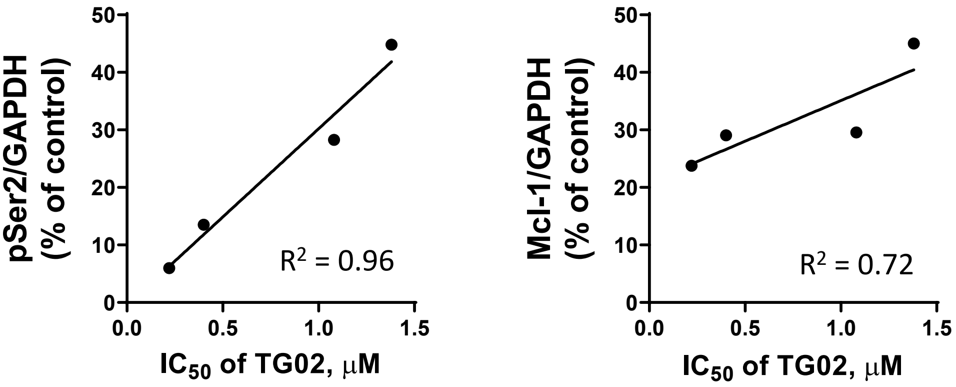

Supplemental Figure 2. TG02 IC_{50}s correlated to the inhibition of RNA pol II phosphorylation and reduction of Mcl-1.

Phospho Ser2-pol II and Mcl-1 level levels (at 1 μM TG02) were quantitated from the immunoblots in Fig 3, normalized to GAPDH, calculated as percentage of controls and correlated to the IC_{50}s of TG02. R squared values were generated by linear regression with the Prism software.

Chen, R et al., Supplemental Fig. 3

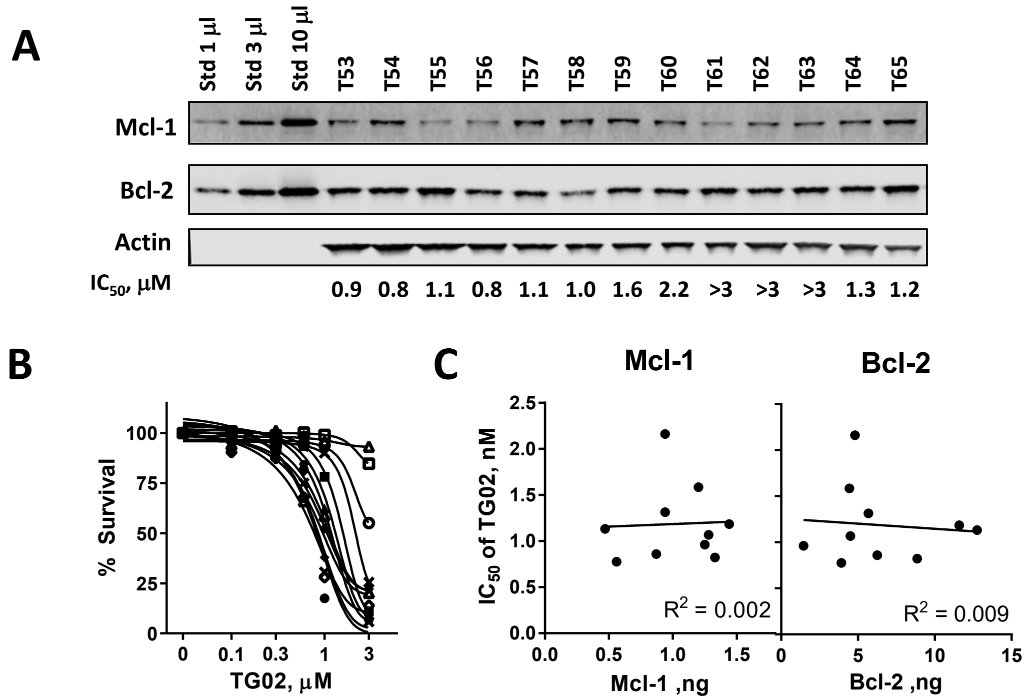

Supplemental Figure 3. Sensitivity to TG02 does not correlate with the basal expression of Mcl-1 or Bcl-2 in the CLL cells. A. Quantitation of basal Mcl-1 and Bcl-2 expression in the CLL cells. CLL cells lysates were collected before TG02 incubation and subjected to immunoblotting along with Mcl-1(Abcam, Cambridge, MA) and Bcl-2 standard proteins (std) (Sigma Aldrich Inc., St. Louis, MO). The amount of standard protein used are 0.5, 1.5 and 5 ng for Mcl-1 , and 2.5, 7.5 and 25 ng for Bcl-2. The bands were quantitated and the amount of Mcl-1 and Bcl-2 (in 20 μg of total protein) were calculated from the standard curve. The IC_{50} values are marked at the bottom of the blots. **B.** The dose response curves of TG02 induced cell death in the CLL samples. CLL cells were incubated with increasing concentrations of TG02 for 24 h and viability were analyzed by Annexin V/PI double staining. **C**. The IC_{50}s of TG02 do not correlate to the basal expression of Mcl-1 or Bcl-2. The IC_{50} of TG02 were plotted against the expression levels of Mcl-1 or Bcl-2. The three samples of which the IC_{50}s were out of dose range were removed from the correlation study. None of the correlations were significant according to Spearman analysis. R squared values were generated by linear regression.

Chen, R et al., Supplemental Fig. 4

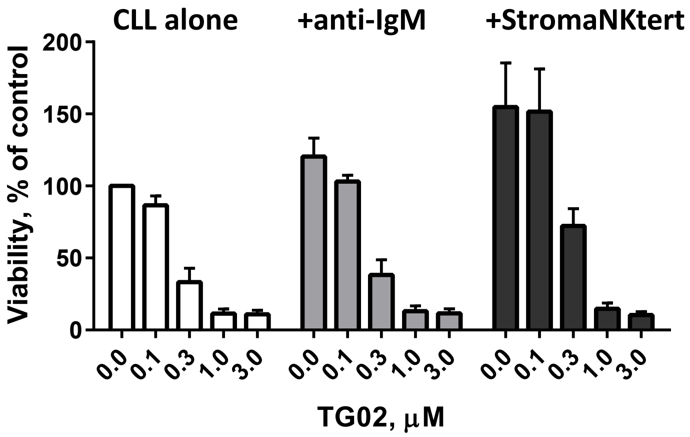

Supplemental Figure 4. TG02 remains active in cell culture conditions mimicking CLL in vivo microenvironment. CLL cells were cultured in RPMI media supplemented with 10% FBS, either alone, or at the presence of anti-IgM or co-culture with StromaNKtert cells. The cells were incubated with increasing concentrations of TG02 and cell viabilities were analyzed by Annexin V/PI double staining at the end of the 24 h incubation. Data represents mean +/- SE of four CLL samples.

Chen, R et al., Supplemental Fig. 5

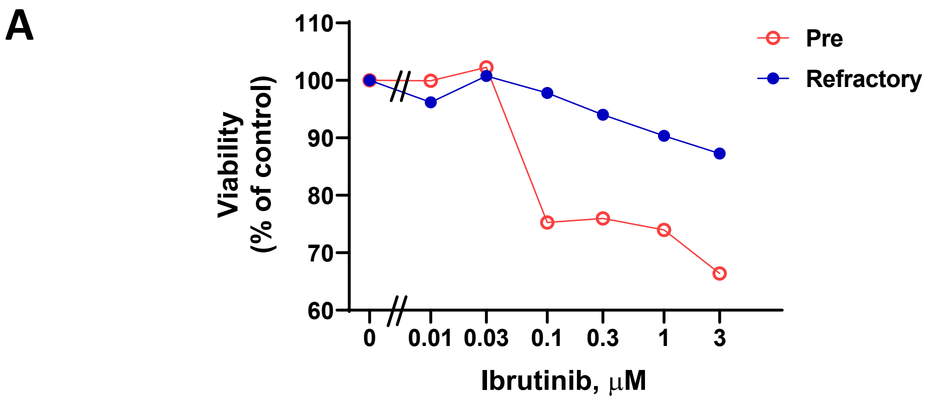

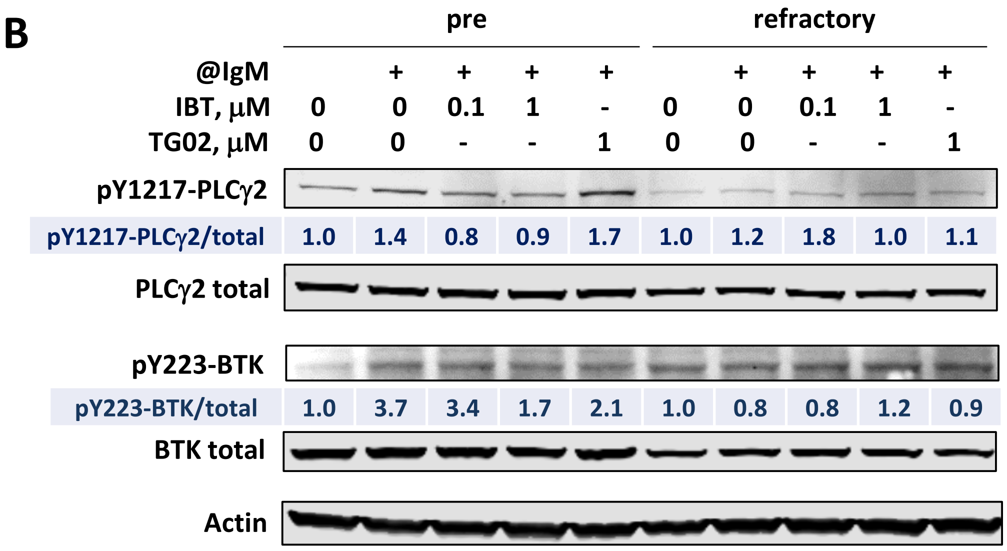

Supplemental Figure 5. CLL cells collected from the patient who was progressing on ibrutinib treatment were less responsive to ibrutinib *in vitro*. A. CLL cells were collected from the same patient prior to the initiation of ibrutinib therapy (pre) and at the time of progression (refractory). Cell viabilities were compared at 24 hours after incubation with increasing concentrations of ibrutinib. **B**. Comparing the effect of ibrutinib on the its cellular targets by immunoblotting in cells collected from the same patient prior to the initiation of ibrutinib therapy (pre) and at the time of progression (refractory). The phosphorylated and total protein were quantitated and presented as levels compared to controls without @IgM.
